# Diagnostic Performance of SARS-CoV-2 Rapid Antigen Test in relation to RT-PCR C_q_ Value

**DOI:** 10.1155/2022/9245248

**Published:** 2022-05-10

**Authors:** Dipendra Kumar Mandal, Bibek Raj Bhattarai, Sushant Pokhrel, Mandira Chhusyabaga, Parmananda Bhandari, Mahendra Prasad Bhatt, Sujan Babu Marhattha

**Affiliations:** ^1^Department of Laboratory Medicine, Manmohan Memorial Institute of Health Sciences, Kathmandu, Nepal; ^2^Sukraraj Tropical and Infectious Disease Hospital, Teku, Kathmandu, Nepal; ^3^Central Department of Bio-Technology, TU, Kathmandu, Kirtipur, Nepal; ^4^Department of Molecular Lab, Nepal Lab House, Kathmandu, Nepal

## Abstract

**Background:**

Early detection of the SARS-CoV-2 is crucial for both the improvement of turnaround time and limiting the spread of the virus in the community. Thus, this study aims to establish rapid antigen tests as an effective diagnostic tool to improve the testing strategies of COVID-19 diagnosis.

**Methods:**

A laboratory based cross-sectional study was performed on the patients that visited Sukraraj Tropical and Infectious Disease Hospital (STIDH) in Kathmandu, Nepal, from November 2020 to January 2021. A total of 213 nasopharyngeal swabs were collected from both symptomatic and asymptomatic patients for rapid antigen test, followed by RT-PCR assay as reference test for confirmation of COVID-19. A standard questionnaire was administered to collect other information from patients. Data were collected and analyzed using SPSS version 20.

**Results:**

Out of 213 individuals, 75 tested positive in Ag-RDT test, while 118 tested positive for SARS-CoV-2 RNA genome via Real time PCR assay. The overall diagnostic performance of Ag-RDT showed 63.6% sensitivity and 97.9% specificity. The diagnostic accuracy of Ag- RDT was 78.9% with *κ* value 0.590, showing moderate agreement with RT-PCR. Significant difference (*p* value <0.001) was observed between Ag- RDT^+^ and Ag- RDT^−^ results when compared to C_q_ values obtained from RT- PCR.

**Conclusion:**

The promising performance of Ag-RDT renders it useful as screening tool alongside RT-PCR to reduce transmission via improving contact tracing, implementation of local mitigation strategies, and refining existing testing protocol for diagnosis of COVID-19.

## 1. Introduction

SARS-CoV-2, a member of the family *Coronaviridae,* is a large, spherical, enveloped virus with positive sense single-stranded RNA genome ranging 25–32 kb. [1]. The virus contains about four structural proteins namely spike glycoprotein (S-protein), membrane protein (M-protein), envelope protein (E-protein), and nucleocapsid protein (N-protein). Polyprotein 1a and polyprotein 1ab encoded by ORF1a and ORF1ab comprise nonstructural proteins or NSPs, that is, NSP1-NSP11 and NSP12-NSP16, respectively [2].

Ever since its discovery in Wuhan, China, in December 2019, it has been causing pandemic till today [3]. The virus originally known as 2019-nCoV, was renamed by International Committee of Taxonomy of Viruses (ICTV) to SARS-CoV-2 [4]. WHO declared a global pandemic on March 11, 2020, due to rapid transmission rate and the infection severity of SARS-CoV-2 [5]. SARS-CoV-2 has an incubation period of 5.2 days, with transmission occurring 1–3 days before symptoms appear [6]. Progression of COVID-19 in advanced and severe cases may lead to severe pneumonia, multiple organ dysfunction, and even death in people with comorbidities [7]. First case presentation of COVID-19 in Nepal was officially reported in a male of age 32 on January 24, 2020 [8]. The newly discovered virus has created problems for its diagnosis, prognosis as well as treatment of this disease.

Various tests for COVID-19 diagnosis include Nucleic Acid Amplification Testing (NAAT) such as RT-PCR, Computed Tomography scan (CT-scan), Protein testing via ELISA, Point of Care Tests (POCT) such as lateral flow assays, use of biosensors, etc. [9]. Currently, the recommended gold standard assay for COVID-19 diagnosis is RT-PCR which involves conversion of SARS-CoV-2 RNA into complementary DNA (cDNA) using reverse transcription followed by specific region amplification of cDNA [10, 11]. C_q_ values obtained from RT-PCR can be an indirect, semiquantitative measurement of viral load and can be considered to be of greater value in determining the infectiousness [12]. As per MIQE guidelines, the terms *cut-off point (C*_*p*_*), take-off point (TOP)*, and *cycle threshold (C*_*t*_) all represent the same meaning and are standardized as *Quantification cycle (C*_*q*_) [13].

Viral extraction and RT-PCR processing requires aseptic sample collection technique, specialized laboratory setup and highly skilled health professional specialist to analyze and interpret the obtained result [14]. RT-PCR processing takes a long time for test completion which in turn increases the overall turnaround time causing problems in mass screening, contact tracing, and disease surveillance [15, 16]. Rapid point-of-care antigen test (Ag-POCT) is a qualitative test that is based on a principle of lateral flow assay in which SARS-CoV-2 antigen is detected in a patient's sample following color change in the kit. N-antigen is detected by most of the Ag-RDT due to its relative abundance and genomic conservation [17]. It has been approved by WHO in low- and middle-income countries with under-resourced laboratories and may be effective in detecting antigenic virus particles in a short time period, and aid in diagnosis of early infection [18, 19].

Hence, this study attempts to demonstrate the effective use of rapid antigen test to decrease the turnaround time for effective COVID-19 diagnosis and its use as screening test to reduce transmission at the local level.

## 2. Methods

This laboratory-based cross-sectional study was performed in Sukraraj Tropical and Infectious Disease Hospital (STIDH), Kathmandu, Nepal, in collaboration with Manmohan Memorial Institute of Health Sciences (MMIHS), Kathmandu, Nepal during the period of 3 months (November 2020 to January 2021).

### 2.1. Inclusion and Exclusion Criteria

After obtaining written informed consent, both symptomatic and asymptomatic individuals were selected for the study. Patients of all age groups, referred by physicians of STIDH and contact tracing with symptoms, were regarded as symptomatic cases. Individuals suspected without any symptoms via contact tracing were referred as asymptomatic individuals.

Specimens collected from previously positive patients, during their follow-ups within the study period, were excluded from the study.

### 2.2. Experimental Protocol

For SARS-CoV-2 detection, specimens such as nasopharyngeal swab/throat swab were collected in viral transport medium (VTM) (SANLI medical, China) using aseptic technique. These swab samples were immersed in 2 ml VTM and sent to molecular lab, STIDH. Further processing was performed aseptically in class II A_2_biosafety cabinet.

Each individual specimen was initially screened for SARS-CoV-2 N-Ag using a rapid antigen test (Espline®, Japan) based on the principle of lateral flow assay. Briefly, nasopharyngeal swab specimens were immersed into the sample extraction tube containing the extraction buffer. Applicator tip was inserted into the buffer and the tube was left to stand for 5 minutes. Then, two drops of this sample solution was applied on the sample zone of the kit. Immediately, the button was pressed in order to start the assay reaction and detect N Ag. Result was interpreted within 30 mins. Visually observed two blue lines of reference (R) and test (T) were interpreted as positive test result or presence of N Ag. For negative COVID-19 antigen result, only the reference (R) line can be visually seen without a blue line in test (T). If no line was observed in both Reference and Test, the test was considered as invalid.

RT-PCR was used to detect SARS-CoV-2 RNA genome. First, nucleic acid from the sample was extracted as per manufacturer's guideline (Zybio Inc., China). Then a template was added to the prepared mater-mix (Shenzhen Unimedica Technology, China). The primer set and FAM labeled probe was designed for SARS-CoV-2 *ORF1ab gene* detection, while VIC labeled probe for detection of SARS-CoV-2 N-gene. Human *RNase P* gene labeled with CY5 extracted simultaneously with test sample acted as an internal control to validate nucleic acid extraction procedure and reagent integrity. Result was reported as positive when C_q_ ≤ 38 with S-shaped amplification curve was obtained and reported as negative when null C_q_ or C_q_ = 40 was observed. The detection limit was 200 copies/ml.

### 2.3. Statistical Analysis

Data were analyzed using IBM SPSS version 20.0 (IBM Corp., Armonk, NY, USA). Shapiro–Wilk normality test was applied to obtained data for normality distribution. Mann–Whitney *U* test was used to compare RT-PCR C_q_ values between Ag-positive and Ag-negative test results.

Continuous variables were interpreted as median and interquartile range. Categorical variables were reported in numbers, percentages, and 95% confidence Intervals. Cohen's kappa coefficient (*κ*) was used to assess the agreement between RT-PCR and antigen tests. *κ* value interpretations were categorized as follows: ≤0 is no agreement, 0.01–0.20 is none to slight, 0.21–0.40 is fair, 0.41–0.60 is moderate, 0.61–0.80 is substantial, and 0.81–1.00 as almost perfect agreement [20].

## 3. Results

Among 213 study population, the median age was 35 years (IQR 27–46.5). Male and female subjects were 67.1% (*n* = 143/213) and 32.9% (*n* = 70/213), respectively. Among samples tested for possible SARS-CoV-2 infection by rapid antigen test method, 36.2% (*n* = 77/213) indicated positive results and 63.8% (*n* = 136/213) showed negative test results. All the test results were then confirmed by real time RT-PCR assay, where 55.4% (*n* = 118/213) tested positive, while 44.6% (*n* = 95/213) tested negative for SARS-CoV-2 viral RNA genome as shown in Table 1.

Among 118 RT-PCR positive cases, 31.3% (*n* = 37) were female and 68.7% (*n* = 81) were male with 55.4% period prevalence as presented in Table 2.

**Figure 1:**
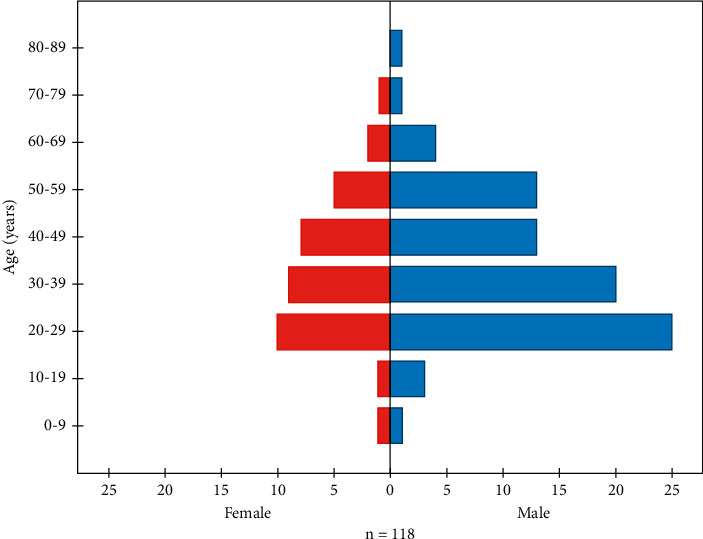
Age distribution of RT-PCR positive cases by gender, demonstrating high SARS-CoV-2 infection among both male and female subjects of age range 20–29 years followed by the age group ranging 30–39 years.

Figure 1 shows age distribution of RT-PCR positive cases by gender, demonstrating high SARS-CoV-2 infection among both male and female subjects of age range 20–29 years followed by the age group ranging 30–39 years.

**Figure 2:**
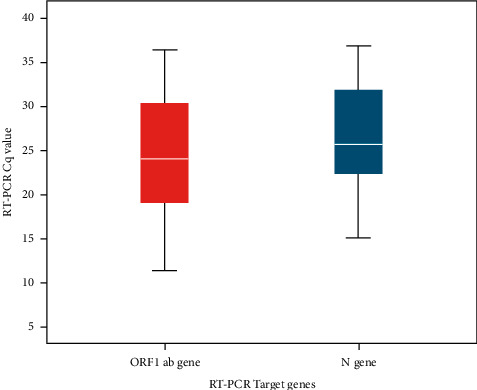
Cq value distribution of RT-PCR target genes. Middle horizontal line inside the box denotes the median. The boxes represent interquartile range (lower, 1st quartile and upper, 3rd quartile). The lower and upper whiskers represent minimum and maximum C_q_ values, respectively.

RT-PCR consists of dual target genes.  Figure 2 illustrates box plot graphs for C_q_ value distribution of RT-PCR target genes. Middle horizontal line inside the box denotes the median. The median C_q_ value for *ORF1ab gene* and *N gene* were 24.05 and 25.69, respectively. For *ORF1ab gene*, maximum and minimum values were 36.42 and 11.33 , respectively; for *N gene* maximum value was 36.89, and minimum value was 15.03, as represented by whiskers in the figure. As for interquartile range represented by the box in the graph as lower 1^st^ quartile and upper 3^rd^ quartile, *ORF1ab* gene has IQR 18.98–30.34, while for *N gene* IQR spans from 22.28–32.96.

Out of 118 RT-PCR positive subjects, the rapid antigen test correctly classified 75 individuals as having the disease. A total of 93 cases were reported by both antigen test and RT-PCR as negative were considered true negatives. Discordant results were obtained between RT-PCR and antigen test, that is, 2 false positives and 43 false negatives (Table 3). A total of 60.4% (26/43) patients with false negative results had a Cq value of >30.

Table 4 summarizes the characterization of diagnostic performance of rapid antigen test. The antigen test showed sensitivity and specificity of 63.6% (CI 54.7–71.9%) and 97.9% (CI 93.6–99.6%), respectively. With 55.4% being the period prevalence of COVID-19 within the tested population, positive predictive value and negative predictive value were 97.4% (CI 92.2–99.6%) and 68.4% (CI 60.3–75.8%), respectively. Agreement analysis via Cohen's kappa showed *κ* coefficient value, 0.590 (CI 49.2–68.8%), *p* < 0.005 demonstrating moderate agreement between RT-PCR and Ag-RDT.

C_q_ values of individual genes were stratified into four groups, that is, <20, 20– < 25, 25– < 30, and 30– < 37. The antigen test results were compared to that of RT-PCR positive results accordingly. Sensitivity in clinical samples with C_q_ < 20 ranged 85.7–88.9%, between 82.1 and 87.2% in C_q_ 20– < 25 and 58.3–71.0% in C_q_ 25– < 30, respectively. Poor performance was observed in C_q_ value > 30, that is, 30–37 with sensitivity as low as 20.0–20.6% (Table 5).

**Figure 3:**
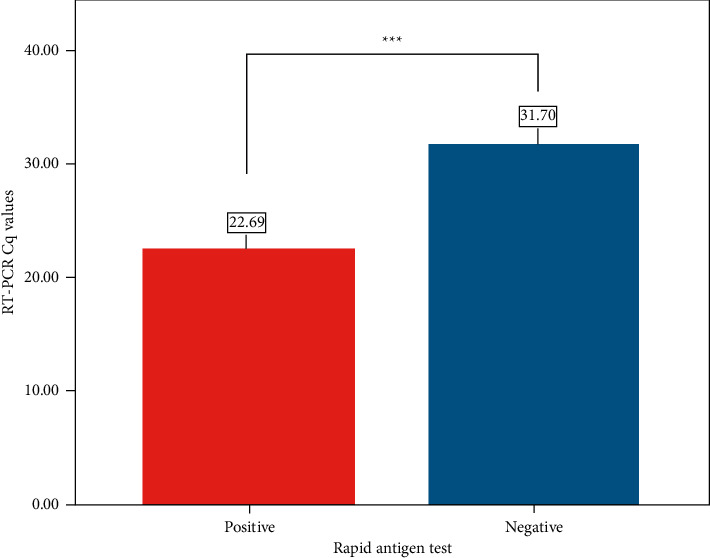
Positive and negative Ag-RDT test results in relation to RT-PCR C_q_ values. ^*∗∗∗*^ represents *p* value <0.001.

Figure 3 demonstrates the test result of rapid antigen test in relation to RT-PCR C_q_ C_q_ value in Ag-RDT^+^ was 22.69, while in Ag-RDT^−^ case the C_q_ value was much higher at 31.70. The overall differences between the two groups, that is, RT-PCR^+^/Ag-RDT^+^ and RT-PCR^+^/Ag- RDT^−^ were significant with *p* value < 0.001.

**Figure 4:**
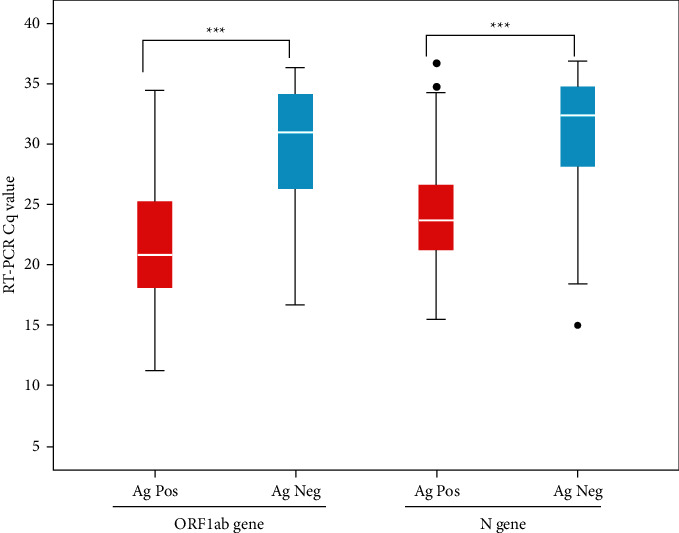
Distribution of ORF1ab gene and N gene C_q_ value in positive and negative rapid antigen test results. Middle horizontal line inside the box denotes the median. The boxes represent interquartile range (lower, 1st quartile and upper, 3rd quartile). The lower and upper whiskers represent minimum and maximum C_q_ value distribution, respectively. ^*∗∗∗*^ represents *p* value <0.001.

RT-PCR C_q_ values of each target gene were studied independently to further demonstrate the performance of antigen test results. For the *ORF1ab gene*, the cases considered positive by Ag- RDT had a median C_q_ value of 20.84 (IQR 25.33–18.03), while the cases considered negative had a median value of 30.97 (IQR 25.98–34.23). For *N gene*, the cases detected by Ag- RDT had a median C_q_ value of 23.71 (IQR 21.22–26.72), and the cases missed by Ag-RDT had a median C_q_ value of 32.43 (IQR 27.55–34.87).  Figure 4 demonstrates the box plot graph of *ORF1ab gene* and *N gene* C_q_ value in positive and negative rapid antigen test results. The differences between the two group, that is, RT-PCR^+^/Ag-RDT^+^ and RT-PCR^+^/Ag- RDT^−^ were significant for individual genes with *p* value < 0.001.

## 4. Discussion

Nucleic acid amplification test (NAAT) is used as the gold standard test for diagnosis of COVID-19. RT-PCR is a widely used molecular technique for detection of SARS-CoV-2 viral genome. Despite the increased ability of RT-PCR to accurately diagnose infected individuals, its delay in turnaround time during pandemic caused stress in mass population screening and disease surveillance. Adoption of biosensor to detect COVID-19 has also been widely popular in this scenario. Jing Wang along with his researchers contributed to the development of an optical based sensor to detect SARS-CoV-2 RNA from patients [21]. Antigen test, based on lateral flow assay is another important tool to diagnose the active infection which gives results within minutes, and is easy to interpret.

The study conducted in China illustrated higher SARS-CoV-2 infection in males, 63.8% rather than in females, 36.2% which is in correspondence with our study analysis which demonstrated 68.7% and 31.3% male and female infection rate, respectively [7]. Our study also demonstrated the more infected age group, 20–29 years followed by age group 30–39 years, corresponding with the results obtained from the study conducted by Sharma et al. illustrating males of age group 21–30 years to be more infected [22]. In contrast, one study reported a higher incidence rate among females [23], whereas some studies suggest higher infection in the age group 30–39 years followed by 20–29 years of age [24].

The reported sensitivity and specificity of Ag-RDT by the product manufacturer were 80% and 100%, respectively. In our study, the overall sensitivity and specificity of rapid antigen test were found to be lower than the manufacturer, that is, 63.6% (75/118) and 97.9% (93/95), respectively, but showed almost similar results of specificity as recommended by WHO, that is, ≥97%, but less sensitivity, that is, ≤80% [25]. In contrast, Aoki et al. observed lower diagnostic performance of rapid antigen test (sensitivity, 39.7% and specificity, 97.0%) than our findings. The performance of rapid antigen test depends upon site of sample collection, sample handling, viral load, and C_q_ value along with antigen extraction process and antigen kit used [3, 26, 27].

In our study, discrepancy results were obtained between antigen test and RT-PCR with 2 false positives and 43 false negatives. Although still unclear about the cause of result discrepancy, 60.4% (26/43) of false negative cases in our study had C_q_ ≥ 30 which corresponds to low viral load explaining the false negative results obtained [28]. As per Robert Koch Institute, the individuals with C_q_ > 30 can be considered noncontagious [29]. Since, the detection by Ag-RDT does not need the gene amplification step, unlike PCR which requires amplification of nucleic acid in order to detect the presence of viral RNA genome [30], this may be the reason for major discrepancy between test results of RT-PCR and rapid antigen tests [31].

The diagnostic accuracy from the findings of our data was found to be 78.9% with a Cohen's weighted kappa value 0.590 displaying moderate agreement between Ag-RDT and RT-PCR, which is similar to the findings obtained by Kohmer et al. during his study [32]. In contrast, a study observed that kappa value 0.859 showed almost a strong agreement between the tests [33]. The reason for this difference is due to the fact that kappa value is highly influenced by data distribution and presence of bias between observers [34, 35].

C_q_ value of lower range had higher chance of positive rapid antigen test result that was reported by Routsias et al. [36]. From our analysis, the overall C_q_ value median was higher in negative rapid antigen test (31.70) in comparison to C_q_ values in positive antigen test (22.69) which suggests that the probability of Ag-RDT to give true positive cases increase when C_q_ value is < 25, while probability of getting negative result increases when C_q_ value is > 30. Higher C_q_ value in negative rapid antigen test was also observed by Young et al. [37]. Our study data also indicated that the significant true positive rate decreased with subsequent increase in C_q_ value. Comparable findings were obtained in a study conducted in Japan by Takeda et al. [38]. Another study conducted by Kahn et al. also ended up with the same conclusion due to the fact that high C_q_ value is indicative of lower viral load [39], subsequently lowering the performance of rapid antigen test [40].

Statistically significant results were obtained between positive and negative rapid antigen test when compared with C_q_ values of individual target genes obtained from RT-PCR. A study conducted by Tregiarri et al. too demonstrated the statistically significant result (*p* < 0.001) when Ag-RDT data were compared to that of C_q_ values obtained [41].

Our study also had weaknesses and limitations of its own which would have been proven beneficial for the study. Since the clinical data were not obtained regarding the patient's previous health status or prior infection with other pathogens, cross reactivity could not be excluded. Quantitative estimation of RNA genome and virus culture could not be performed due to shortage of proper resources, although it could have been useful in precisely analyzing the diagnostic performance of rapid antigen test.

## 5. Conclusion

The diagnostic performance of the rapid antigen test is in relation to RT-PCR C_q_ value: sensitivity of Ag-RDT is indirectly proportional to C_q_ value. Ag-RDT performance when compared to RT-PCR has decreased sensitivity but comparable specificity. Despite low diagnostic sensitivity, rapid results within minutes, inexpensiveness, and ease of result interpretation makes Ag-RDT valuable in reducing transmission by facilitating rapid isolation, contact tracing in community. The Ag-RDT performance seems to be promising and can be used as a rapid screening tool in patients with high viral load alongside RT-PCR to further improve the testing strategies for diagnosis of COVID-19.

## Figures and Tables

**Table 1:** Characteristics of study population.

Characteristics	Result
Age Median (IQR)	35.00 (28–46.5)
Gender Male (%) Female (%)	143 (67.1%) 70 (32.9%)
RT-PCR assay Positive (%) Negative (%)	118 (55.4%) 95 (44.6%)
Antigen test Positive (%) Negative (%)	77 (36.2%) 136 (63.8%)

IQR, interquartile range; RT-PCR, reverse transcriptase polymerase chain reaction.

**Table 2:** Characteristics of RT-PCR confirmed COVID-19 cases.

Characteristics	Result
Period prevalence	55.4%
Gender Male (%) Female (%)	81 (68.7%) 37 (31.3%)

**Table 3:** Contingency table showing the PCR and antigen test results.

		PCR assay		Total antigen result
		Positive	Negative	
Antigen tests	Positive	75	2	77
	Negative	43	93	136
Total RT-PCR result		118	95	213

**Table 4:** Overall diagnostic performance evaluation of rapid antigen test.

Performance	Result
Sensitivity	63.6% (95% CI 54.7–71.9%)
Specificity	97.9% (95% CI 93.6–99.6%)
Positive likelihood	30.29 (95% CI 7.61–119.77)
Negative likelihood	0.37 (95% CI 0.293–0.473)
Positive predictive value	97.4% (95% CI 92.2–99.6%)
Negative predictive value	68.4% (95% CI 60.3–75.8%)
Accuracy	78.9%
Kappa value (*κ* value)	1.590 (95% CI 0.492–0.688), *p* < 0.005

**Table 5:** Sensitivity of rapid antigen test in each Cq values stratified cut-offs.

C_q_ value	Target gene	N	Sensitivity (%)
<20	*ORF1ab* gene	36	88.9
	*N* gene	14	85.7

20– < 25	*ORF1ab* gene	28	82.1
	*N* gene	39	87.2

25– < 30	*ORF1ab* gene	24	58.3
	*N* gene	31	71.0

30– < 37	*ORF1ab* gene	30	20.0
	*N* gene	34	20.6

## Data Availability

All the data generated during this study are presented. The primary raw data will be made available to interested researchers upon request to the corresponding author.

## Abbreviations

Ag-RDT: Rapid antigen detection test
COVID-19: Coronavirus disease 2019
Cq: Quantification cycle
FN: False negative
FP: False positive
MIQE: Minimum information for publication of quantitative real-time pcr experiments
N gene: *Nucleocapsid* gene
N-Ag: Nucleocapsid antigen
nCoV: Novel coronavirus
NPV: Negative predictive value
ORF1ab: Open reading frame 1a/b
PPV: Positive predictive value
RT-PCR: Real-time polymerase chain reaction
SARS-CoV-2: Severe acute respiratory syndrome coronavirus 2
TN: True negative
TP: True positive
WHO: World Health Organization
*κ* value: Kappa coefficient value.
